# Critical brain wave dynamics of neuronal avalanches

**DOI:** 10.3389/fphy.2023.1138643

**Published:** 2023-02-22

**Authors:** Vitaly L. Galinsky, Lawrence R. Frank

**Affiliations:** 1 Center for Scientific Computation in Imaging, University of California, San Diego, San Diego, CA, United States,; 2 Center for Functional MRI, University of California, San Diego, San Diego, CA, United States

**Keywords:** non-linear waves, critical exponent, Hamiltonian system, neuronal avalanches, critical dynamics

## Abstract

Analytical expressions for scaling of brain wave spectra derived from the general non-linear wave Hamiltonian form show excellent agreement with experimental “neuronal avalanche” data. The theory of the weakly evanescent non-linear brain wave dynamics reveals the underlying collective processes hidden behind the phenomenological statistical description of the neuronal avalanches and connects together the whole range of brain activity states, from oscillatory wave-like modes, to neuronal avalanches, to incoherent spiking, showing that the neuronal avalanches are just the manifestation of the different non-linear side of wave processes abundant in cortical tissue. In a more broad way these results show that a system of wave modes interacting through all possible combinations of the third order non-linear terms described by a general wave Hamiltonian necessarily produces anharmonic wave modes with temporal and spatial scaling properties that follow scale free power laws. To the best of our knowledge this has never been reported in the physical literature and may be applicable to many physical systems that involve wave processes and not just to neuronal avalanches.

## 1 Introduction

The complexity of oscillatory and wave patterns across a wide range of spatial and temporal scales of brain activity results in multiple independent models for these activity patterns. The standard view of brain electromagnetic activity classifies this activity into two significant but essentially independent classes. The first class includes a variety of the oscillatory and wave-like patterns that show relatively high level of coherence across a wide range of spatial and temporal scales [3]. The second class focusses on the asynchronous, seemingly incoherent spiking activity at scales of a single neuron and often uses various *ad hoc* neuron models [4–8] to describe this activity. Linking these two seemingly disparate classes to explain the emergence of oscillatory rhythms from incoherent activity is essential to understanding brain function and is typically posed in the form using the construct of networks of incoherently spiking neurons [9–11].

Coherent macroscopic behavior arising from seemingly incoherent microscopic processes naturally suggests the influence of critical phenomena, a potential model from brain activity that was bolstered by the experimental discovery of the “neuronal avalanches” [12, 13] where both spatial and temporal distributions of spontaneous propagating neuronal activity in 2D cortex slices were shown to follow scale-free power laws. This discovery has generated significant interest in the role and the importance of criticality in brain activity [14–20]. Crucial events, as a manifestation of criticality, have been discussed in [21] using Diffusion Entropy Analysis [22]. It was also hypothesized that an existence of crucial events facilitates information transmission in various states of brain functioning [23, 24].

Although the precise neuronal mechanisms leading to the observed scale-free avalanche behavior is still uncertain after almost 20 years since their discovery, the commonly agreed upon paradigm is that this collective neuronal avalanche activity represents a unique and specialized pattern of brain activity that exists somewhere between the oscillatory, wave-like coherent activity and the asynchronous and incoherent spiking. Central to this claim of neuronal avalanches as a unique brain phenomena is that they do not show either wave-like propagation or synchrony at short scales, and thus constitute a new mode of network activity [12, 13] that can be phenomenologically described using the ideas of the self-organized criticality [25, 26], and extended to the mean-field theory of the self-organized branching processes (SOBP) [27–29].

However, despite the success of the SOBP theory in describing neuronal avalanche statistical properties, i.e., replicating the power law exponents based on the criticality considerations, the SOBP theory provides no explanation about the physical mechanisms of the critical behavior and its relationship to the development of the observed collective neuronal “avalanche” behavior. Because similar statistics can result from several mechanisms other than critical dynamics [30–32], it is essential to have a physical model that explains the relationship between the statistical properties and the existence, if any, of critical neural phenomena arising from the actual collective behavior of neuronal populations. While it is generally accepted in that some form of critical phenomena is at work, this has led to the presupposition of *ad hoc* descriptive models [33–36] that exhibit critical behavior, but provide no insight into the actual physical mechanisms that might produce such critical dynamics. It has been suggested that the brain can be at the edge of a synchronization phase transition [36–38], but the usual agreement is that avalanches belong to the mean-field directed percolation universality class, which does not seem to be compatible with a synchronization transition, as synchronization transitions do not fulfill spatial correlations observed in experiments, and the exponents tend to differ from directed percolation ones [20].

In this paper we show that the above important observational phenomenon, the so-called “neuronal avalanches”, which have been noted and studied for almost 2 decades, can be naturally explained by the wave activity model. Our recently described theory of weakly evanescent brain waves (WETCOW) originally developed in [1, 2] and then reformulated in a general Hamiltonian framework [39] provides a physical theory, based on the propagation of electromagnetic fields through the highly complex geometry of inhomogeneous and anisotropic domain of real brain tissues, that explains the broad range of observed seemingly disparate brain wave characteristics. This theory produces a set of non-linear equations for both the temporal and spatial evolution of brain wave modes that includes all possible non-linear interaction between propagating modes at multiple spatial and temporal scales and degrees of non-linearity. This theory bridges the gap between the two seemingly unrelated spiking and wave “camps” as the generated wave dynamics includes the complete spectra of brain activity ranging from incoherent asynchronous spatial or temporal spiking events, to coherent wave-like propagating modes in either temporal or spatial domains, to collectively synchronized spiking of multiple temporal or spatial modes.

We further demonstrate that the origin of these “avalanche” properties emerges directly from the same theory that produces this wide range of activity and does not require one to posit the existence of either new brain activity states, nor construct analogies between brain activity and *ad hoc* generic “sandpile” models. Both temporal and spatial scaling expressions analytically derived from non-linear amplitude/phase evolutionary equations show excellent agreement with the experimental neural avalanche probability spectra reproducing not only general average power law exponent values and falloffs in the vicinity of the critical point, but also finding some very subtle but nevertheless clearly experimentally evident fine details, like bumps in the transition region at the edge of the power leg of the spatial probability spectra. Overall, the quantitative theoretical analysis presented in the paper clearly shows the relevance of the wave Hamiltonian framework to the neuronal avalanche dynamics and suggests that instead of relying on clever but *ad hoc* analogies between live brain tissues and lifeless loose sand piles often used to construct a phenomenological statistical description of the scaling exponents, both the origin of the critical phenomena and the physics behind the neuronal avalanches can be understood from the same non-linear wave dynamics that is responsible for the wide range of activities in the brain tissue, ranging from the linear coherently propagating waves to the non-linear incoherent asynchronous spiking, including in between the peculiar power law-like coherence of the neuronal avalanches.

We emphasize that although the general WETCOW theory describes complex interactions between modes, the explanation for neuronal avalanches and their attendant scaling properties presented in this paper are based on the analysis of a *single wave mode* with completely arbitrary set of mode parameters. This includes arbitrary amplitude, phase, frequency, and criticality. No interaction between modes, except a general form of the third order non-linearity that characterizes anharmonicity of the non-linear wave modes due to non-resonant interaction of the linear modes, is needed to derive the scaling result. Thus a key result of this paper is the demonstration that neuronal avalanches and their attendant scaling properties are obtained within the simplest form of the WETCOW theory where mode coupling is ignored, but significantly without the *ad hoc* and physically implausible assumptions typically made that the parameters of all network nodes are either constant and the same for all nodes [36], sometimes even including inter-mode coupling [38], or are generated from some *ad hoc* artificial distributions [40], and require the addition of stochastic noise properties [41], *etc*. This emphasizes generality and importance of our derivation.

## 2 Weakly evanescent brain waves

Beginning from our non-linear Hamiltonian formulation of the WETCOW theory [39], we have for an anharmonic wave mode

(1) $$H^{s}\left(a,a^{†}\right)=\Gamma aa^{†}+aa^{†}\left[\beta_{a}a+\beta_{a^{†}}a^{†}-2\alpha {\left(aa^{†}\right)}^{1/2}\right]$$

where *a* is a complex wave amplitude and *a*^†^ is its conjugate. The amplitude *a* denotes either temporal *a*_*k*_(*t*) or spatial *a*_*ω*_(*x*) wave mode amplitudes that are related to the spatiotemporal wave field *ψ*(*x*, *t*) through a Fourier integral expansions.

(2) $$a_{k}(t)=\frac{1}{2\pi }\overset{\infty }{\underset{-\infty }{\int }}\psi (x,t)e^{-i\left(kx+\omega_{k}t\right)}dx,$$

(3) $$a_{\omega }(x)=\frac{1}{2\pi }\overset{\infty }{\underset{-\infty }{\int }}\psi (x,t)e^{-i\left(k_{\omega }x+\omega t\right)}dt,$$

where for the sake of clarity we use one dimensional scalar expressions for spatial variables *x* and *k*, but it can be easily generalized for the multi dimensional wave propagation as well. The spatiotemporal wave field *ψ*(*x*, *t*) is a superposition of multiple waves, that may include neuronal firings, membrane sub-threshold oscillations, LFPs, *etc*. The frequency *ω* and the wave number *k* of the wave modes satisfy dispersion relation *D*(*ω*, *k*) = 0, and *ω*_*k*_ and *k*_*ω*_ denote the frequency and the wave number roots of the dispersion relation (the structure of the dispersion relation and its connection to the brain tissue properties has been discussed in [1]).

The first term Γ*aa*^†^ in Eq.1 denotes the harmonic (quadratic) part of the Hamiltonian with either the complex valued frequency Γ = *iω* + *γ* or the wave number Γ = *ik* + *λ* that both include a pure oscillatory parts (*ω* or *k*) and possible weakly excitation or damping rates, either temporal *γ* or spatial *λ*. The second anharmonic term is cubic in the lowest order of non-linearity and describes the interactions between various propagating and non-propagating wave modes, where *α*, *β*_*a*_ and $\beta_{a^{†}}$ are the complex valued strengths of those different non-linear processes. As it was shown in [1, 2] the inverse proportionality of frequency and wave number in the dispersion relation 6) results in the third order expressions for the non-resonant coupling between multiple waves.

Distribution of various charges in brain tissue, including free ionic charges (sodium, potassium, etc), bonded macromolecular volume charges, membrane polarization and/or surface charges, *etc*., determines brain electrodynamics. The voltages and currents measured in real brains are produced by those electrodynamic processes that in the most general form can be represented by the Maxwell equations together with state or motion equations for the brain matter, particularly by a generalized Ohm’s law, that describes electro-diffusive flow of charged particles through inhomogeneous media (that may include both concentration and voltage gradients). The neuron action potential itself is nothing more than propagating non-linear electrostatic wave described by the same electrodynamics formalism. A set of derivations that lead to this description was presented in details in [1, 2, 39] and is based on considerations that follow from the most general form of brain electromagnetic activity expressed by Maxwell equations in inhomogeneous and anisotropic medium

$$\nabla \cdot D=\rho ,\;\nabla \times H=J+\frac{\partial D}{\partial t}\;\Rightarrow \;\frac{\partial \rho }{\partial t}+\nabla \cdot J=0.$$

Using the electrostatic potential ***E*** = −∇*ψ*, Ohm’s law ***J*** = ***σ*** ·***E*** (where ***σ*** ≡ {*σ*_*ij*_} is an anisotropic conductivity tensor), a linear electrostatic property for brain tissue ***D*** = *ε****E***, assuming that the scalar permittivity *ε* is a “good” function (i.e. it does not go to zero or infinity everywhere) and taking the change of variables ∂*x* → *ε*∂*x*′, the charge continuity equation for the spatial-temporal evolution of the potential *ψ* can be written in terms of a permittivity scaled conductivity tensor **Σ** = {*σ*_*ij*_/*ε*} as

(4) $$\frac{\partial }{\partial t}\left(\nabla^{2}\psi \right)=-\nabla \cdot \Sigma \cdot \nabla \psi +ℱ,$$

where we have included a possible external source (or forcing) term ℱ. For brain fiber tissues the conductivity tensor **Σ** might have significantly larger values along the fiber direction than across them. The charge continuity without forcing i.e., (ℱ = 0) can be written in tensor notation as

(5) $$\partial_{t}\partial_{i}^{2}\psi +\Sigma_{ij}\partial_{i}\partial_{j}\psi +\left(\partial_{i}\Sigma_{ij}\right)\left(\partial_{j}\psi \right)=0,$$

where repeating indices denote summation. Simple linear wave analysis, i.e. substitution of *ψ* ~ exp(−*i* (***k*** · ***r*** − Ω*t*)), where ***k*** is the wavenumber, ***r*** is the coordinate, Ω = *ω* + *iγ* is the frequency and *t* is the time, gives the following complex dispersion relation:

(6) $$D(\Omega ,k)=-i\Omega k_{i}^{2}-\Sigma_{ij}k_{i}k_{j}-i\partial_{i}\Sigma_{ij}k_{j}=0,$$

which is composed of the real and imaginary components:

(7) $$\gamma \equiv ℑ\Omega =\Sigma_{ij}\frac{k_{i}k_{j}}{k^{2}}\;\omega \equiv ℜ\Omega =-\frac{\partial_{i}\Sigma_{ij}k_{j}}{k^{2}}$$

Although in this general form the electrostatic potential *ψ*, as well as the dispersion relation *D*(Ω, ***k***), describe three dimensional wave propagation, we have shown [1, 2] that in anisotropic and inhomogeneous media some directions of wave propagation are more equal than others with preferred directions determined by the complex interplay of the anisotropy tensor and the inhomogeneity gradient. While this is of significant practical importance, in particular because the anisotropy and inhomogeneity can be directly estimated from non-invasive methods, for the sake of clarity we focus here on the one dimensional scalar expressions for spatial variables x and k that can be easily generalized for the multi dimensional wave propagation as well.

The multiple temporal *a*_*k*_(*t*) or spatial *a*_*ω*_(*x*) wave mode amplitudes can be used to define the time dependent wave number energy spectral density Π_*k*_(*t*) or the position dependent frequency energy spectral density Π_*ω*_(*x*) for the spatiotemporal wave field *ψ*(*x*, *t*) as

(8) $$\Pi_{k}(t)={\left|a_{k}(t)\right|}^{2},{\text{ Π}}_{\omega }(x)={\left|a_{\omega }(x)\right|}^{2},$$

or alternatively we can add additional length or time normalizations to convert those quantities to power spectral densities instead.

The network Hamiltonian form that describes discrete spectrum of those multiple wave modes was presented in [39] as

(9) $$H\left(a,a^{†}\right)=\underset{n}{\sum }\left[H^{s}\left(a_{n},a_{n}^{†}\right)+\underset{m\neq n}{\sum }\left(a_{n}r_{nm}a_{m}^{†}+a_{n}^{†}r_{nm}^{⋆}a_{m}\right)\right]$$

where the single mode amplitude *a*_*n*_ again denotes either *a*_*k*_ or *a*_*ω*_, ***a*** ≡ {*a*_*n*_} and $r_{nm}=R_{nm}e^{i\Delta_{nm}}$ is the complex network adjacency matrix with *R*_*nm*_ providing the coupling power and Δ_*nm*_ taking into account any possible differences in phase between network nodes. This description includes both amplitude ℜ(a) and phase ℑ(a) mode coupling and as shown in [39] allows for significantly unique synchronization behavior different from both phase coupled Kuramoto oscillator networks and from networks of amplitude coupled integrate-and-fire neuronal units.

The third order nature of the theory is of particular interest, and provide the theory with a broad range of applicability. It has distinctly different characteristics than the harmonic oscillator. Of particular importance is the fact that the third order terms become important when wave amplitudes are high enough but only if or until higher order terms are absent or suppressed by some physical mechanism. This suppression becomes significant in incorporating the anisotropic inhomogeneous and resistive nature of brain tissues. An important consequence derived in [1] is that the inverse frequency–wave number proportionality of the linear wave dispersion guarantees that the resonant terms higher than the third order are not important and can be neglected and, at the same time, the non-resonant third order terms (that are typically excluded when compared to the resonant terms) should now be retained resulting in the third order form of Hamiltonian 1). It is our contention, and the subject of future studies, that the anharmonic third order forms 1) and 9) are not brain specific and can be used to describe oscillations and waves in active media abundant in various areas of physics.

Although the Fourier integrals 2 used for expansion of the spatiotemporal wave field *ψ* into a set of wave modes imply presence of a large (actually infinite) number of modes in the network Hamiltonian 9 the derivation of neuronal avalanches is evident even without this generality of this coupling between modes expressed by the coupling parameters *r*_*nm*_, as it was done in [39]. Thus we will consider an ensemble of non-interacting modes, effectively setting *r*_*nm*_ = 0, for the analysis of this paper. But importantly we will *not* make any assumptions about parameters of the single mode Hamiltonian form 1, assuming that all parameters (Γ, *β*_*a*_, $\beta_{a^{†}}$, *α*) are arbitrary and do not carry any mode dependence. This is a non-trivial point worth emphasizing, as it is a departure from the extant literature wherein the *ad hoc*, and physically implausible, assumption of the equivalence of network nodes is made. Therefore, we will proceed with our analysis of a single mode amplitude *a* suppressing all subscripts and indices, and assuming that *a* denotes *a*_*n*_ where *n* may represent either an arbitrary wave number *k* from a range of wave numbers *k*_0_ ≤ *k* ≤ *k*_1_ or an arbitrary frequency *ω* from a range of wave frequencies *ω*_0_ ≤ *ω* ≤ *ω*_1_.

## 3 Single anharmonic mode criticality

An equation for the non-linear oscillatory amplitude *a* then can be expressed as a derivative of the Hamiltonian form

(10) $$\frac{da}{dt}=\frac{\partial H^{s}}{\partial a^{†}}\equiv \Gamma a+\beta_{a^{†}}aa^{†}+\beta_{a}a^{2}-\alpha a{\left(aa^{†}\right)}^{1/2},$$

after removing the constants with a substitution of $\beta_{a^{†}}=1/2{\tilde{\beta }}_{a^{†}}$ and $\alpha =1/3\tilde{\alpha }$ and dropping the tilde. As frequencies and wave numbers for linear waves satisfy the dispersion relation 6), they are related and the same Hamiltonian expression 1) can be used either for temporal *a*_*k*_(*t*) or spatial *a*_*ω*_(*x*) wave amplitudes. Therefore, we note that although (10) is an equation for the temporal evolution, the spatial evolution of the mode amplitudes *a*_*ω*_(*x*) can be described by a similar equation substituting temporal variables by their spatial counterparts, i.e., (*t*, *ω*, *γ*) → (*x*, *k*, *λ*).

Splitting (10) into an amplitude/phase pair of equations using *a* = *Ae*^*iϕ*^, assuming $\beta_{a}={\tilde{\beta }}_{a}e^{-i\delta_{a}}$, $\beta_{a^{†}}={\tilde{\beta }}_{a^{†}}e^{i\delta_{a^{†}}}$, and scaling the variables as

(11) $$A=\gamma \tilde{A},\;t=\frac{\tau }{\gamma },\;\omega =\tilde{\omega }\gamma ,$$

gives the set of equations.

(12) $$\frac{d\tilde{A}}{d\tau }=\tilde{A}+{\tilde{A}}^{2}\left(\beta_{a^{†}}{\text{ cos Ψ}}_{a^{†}}+\beta_{a}{\text{ cos Ψ}}_{a}-\alpha \right)$$

(13) $$\frac{d\phi }{d\tau }=\tilde{\omega }+\tilde{A}\left(-\beta_{a^{†}}{\text{ sin Ψ}}_{a^{†}}+\beta_{a}{\text{ sin Ψ}}_{a}\right)$$

where Ψ_*a*_ ≡ *ϕ* − *δ*_*a*_, $\Psi_{a^{†}}\equiv \phi -\delta_{a^{†}}$.

These equations can further be cast into a more compact form by defining

(14) $$\beta =\left(\begin{array}{c} \beta_{a}\\ \beta_{a^{†}} \end{array}\right),u=\left(\begin{array}{c} e^{i\delta_{a}}\\ e^{i\delta_{a^{†}}} \end{array}\right),v=\left(\begin{array}{c} ie^{i\delta_{a}}\\ -ie^{i\delta_{a^{†}}} \end{array}\right)$$

so that.

(15) $$z_{a}=\beta \cdot u=X_{a}+iY_{a}$$

(16) $$z_{\phi }=\beta \cdot v=X_{\phi }+iY_{\phi }$$

where.

(17) $$R_{a}=\left|z_{a}\right|=\sqrt{X_{a}^{2}+Y_{a}^{2}}$$

(18) $$R_{\phi }=\left|z_{\phi }\right|=\sqrt{X_{\phi }^{2}+Y_{\phi }^{2}}$$

(19) $$\Phi_{a}=\text{arg}\left(z_{a}\right)=\text{arctan}\frac{Y_{a}}{X_{a}}$$

(20) $$\Phi_{\phi }=\text{arg}\left(z_{\phi }\right)=\text{arctan}\frac{Y_{\phi }}{X_{\phi }}$$

whereupon (12) and (13) can be rewritten.

(21) $$\frac{d\tilde{A}}{d\tau }=\tilde{A}+{\tilde{A}}^{2}\left[R_{a}\text{ cos}(\phi -\Phi )-\alpha \right],$$

(22) $$\frac{d\phi }{d\tau }=\tilde{\omega }+\tilde{A}R_{\phi }\text{ cos }\phi ,$$

where Φ = Φ_*a*_ − Φ_*ϕ*_.

A stationary (i.e., *dÃ*/*dτ* = *dϕ*/*dτ* = 0) solution of (21) and (22) can be found from

(23) $$-\frac{R_{\phi }}{\tilde{\omega }}\text{cos }\phi +R_{a}\text{cos }(\phi -\Phi )-\alpha =0,$$

as *ϕ*_*e*_ = *ϕ*_0_ ≡ const and ${\tilde{A}}_{e}=\tilde{\omega }/R_{\phi }\text{ cos }\phi_{0}\equiv \text{const}$. This shows that for *α* > *R*_*a*_ there exist critical values of $\tilde{\omega }$ and *A*_*e*_, where the stationary solution disappears and is replaced by non-linear oscillations, such that.

(24) $${\tilde{\omega }}_{c}=\frac{R_{\phi }\text{ cos }\phi_{c}}{\alpha +R_{a}\text{ cos }\left(\phi_{c}+\Phi \right)},\;{\tilde{A}}_{c}={\tilde{\omega }}_{c}/R_{\phi },$$

(25) $$\phi_{c}=\text{arctan}\left[\frac{R_{a}\text{ sin Φ}}{\sqrt{\alpha^{2}-{\left(R_{a}\text{ sin Φ}\right)}^{2}}}\right],$$

which can also be expressed in terms of critical value of one of the unscaled variables, either *ω* or *γ*

(26) $$\omega_{c}=\gamma {\tilde{\omega }}_{c},\text{ or }\gamma_{c}=\frac{\omega }{{\tilde{\omega }}_{c}},$$

This stationary solution provides the locus of the saddle node on an invariant circle bifurcation point at where the non-linear spiking oscillations occur (as was shown both in [1, 2] and in [39]).

## 4 Effective spiking rate

The effective period 𝒯*_s_* of spiking solutions of (21) and (22) (or its inverse–either the firing rate 1/𝒯*_s_* or the effective firing frequency 2*π*/𝒯*_s_*) can be estimated from (22) by substituting *Ã*_*c*_ for *Ã* (assuming that the change of amplitude *Ã* is slower than the change of the phase *ϕ*) as

(27) $${𝒯}_{s}=\overset{2\pi }{\underset{0}{\int }}\frac{d\phi }{\tilde{\omega }+{\tilde{\omega }}_{c}\text{ cos }\phi }=\frac{2\pi }{\sqrt{{\tilde{\omega }}^{2}-{\tilde{\omega }}_{c}^{2}}},$$

giving the unscaled effective spiking period *T*_*s*_ and the effective firing frequency *ω*_*s*_

(28) $$T_{s}=\frac{{𝒯}_{s}}{\gamma }=\frac{2\pi }{\omega \sqrt{1-\gamma^{2}/\gamma_{c}^{2}}}=\frac{2\pi }{\omega \sqrt{1-\omega_{c}^{2}/\omega^{2}}},$$

(29) $$\omega_{s}=\frac{2\pi }{T_{s}}=\omega \sqrt{1-\omega_{c}^{2}/\omega^{2}},$$

with the periodic amplitude *Ã* reaching the maximum *Ã*_*max*_ 1/(*α* − *R_a_*) and the minimum *Ã*_*min*_ 1/(*α* + *R_a_*) for *dÃ/dτ* = 0 when *ϕ* = Φ and *ϕ* = Φ + *π* respectively.

**FIGURE 1:**
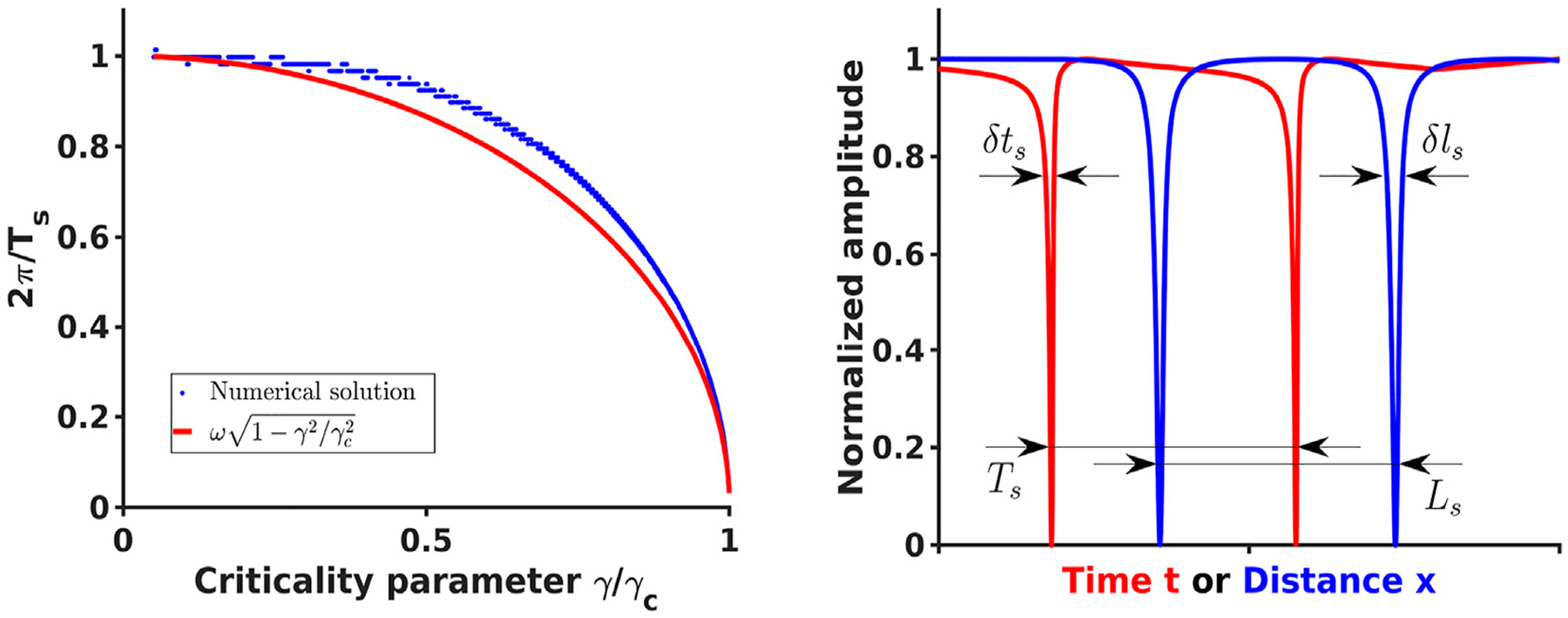
[Left] Comparison of the analytical expression (29) for the effective spiking frequency *ω*_*s*_ = 2*π*/*T*_*s*_ (red) and the frequency estimated from numerical solution of Eq. 21 and Eq. 22 (blue) as a function of the criticality parameter *γ*/*γ*_*c*_. In the numerical solution only *γ* was varied and the remaining parameters were the same as parameters reported in [39] [Right] Spiking solutions for typical parameters producing temporal ((21) and (22), red) and spatial ((38) and (39), blue) spiking profiles where some signal of width *δt*_*s*_ or *δl*_*s*_ was detected and surrounded by quiet area with the total effective period *T*_*s*_ or *L*_*s*_.

The expressions (28) and (29) are more general than typically used expressions for the scaling exponent in the close vicinity |*γ* − *γ*_*c*_|≪ *γ*_*c*_ of the critical point [42–44]. They allow recovery of the correct *T* limits both at *γ* → *γ*_*c*_ with the familiar $T\sim 1/\sqrt{\gamma_{c}-\gamma }$ scaling and at *γ* ~ 0 with the period *T* approaching *T*_0_ as *T* ~ *T*_0_ + *O*(*γ*^2^) ≡ 2*π*/*ω* + *O*(*γ*^2^), where *T*_0_ is the period of linear wave oscillations with the frequency *ω*. In the intermediate range 0 < *γ* < *γ*_*c*_ the expressions (28) and (29) show reasonable agreement (Figure 1) with peak–to–peak period/frequency estimates from direct simulations of system (12) and (13).

## 5 Temporal probability of single spike detection

As the periodic solution of Eq. 21 and Eq. 22 in the 0 ≪ *γ* ≪ *γ*_*c*_ range looks like linear waves at *γ* close to zero, but transforms to spike as *γ* increases, we can approximate the probability of detecting a single spike by a ratio of a spike peak duration (recorded above some threshold) to a total peak-to-peak time. Taking into account that the initial phase of spiking solutions of Eq. 21 and Eq. 22 is a random variable uniformly distributed on [0, 2*π*] interval, the probability that a spike (either positive or the more frequently experimentally reported negative [12, 13]) with duration width *δt*_*s*_ and with the total period between the spikes (*T*_*s*_) will be detected is simply *δt*_*s*_/*T*_*s*_–where the distance between spikes is determined as the time interval needed for 2*π* radian phase change, that is the effective spiking period *T*_*s*_. Assuming initially that the spike width *δt*_*s*_ does not change when approaching the critical point *ω*_*c*_, *δt*_*s*_ can be approximated by some fixed fraction of the linear wave period, i.e., *δt*_*s*_ ~ *π*/*ω*, that gives for the probability density

(30) $$P_{k}^{\left\{\omega \right\}}\left(\omega ,\omega_{c}\right)\sim \omega^{-1}\sqrt{\omega^{2}/\omega_{c}^{2}-1},$$

for every wave mode with the wavenumber *k*. It should be noted that the probability density $P_{k}^{\left\{\omega \right\}}$ has no relation to, and should not be confused, with the frequency energy spectral density Π_*ω*_(*x*) (or with the power spectral density).

Transforming the frequency dependence of the wavenumber spectra $P_{k}^{\left\{\omega \right\}}$ to the temporal domain (*T* = 2*π*/*ω*, *T_c_* = 2*π*/*ω_c_*)

(31) $$\overset{\infty }{\underset{\omega_{c}}{\int }}P_{k}^{\left\{\omega \right\}}\left(\omega ,\omega_{c}\right)d\omega =\overset{T_{c}}{\underset{0}{\int }}P_{k}^{\left\{\omega \right\}}\left(\frac{2\pi }{T},\frac{2\pi }{T_{c}}\right)\frac{2\pi }{T^{2}}dT\;=\overset{T_{c}}{\underset{0}{\int }}P_{k}^{\left\{T\right\}}\left(T,T_{c}\right)dT,$$

gives for the temporal probability density $P_{k}^{\left\{T\right\}}$

(32) $$P_{k}^{\left\{T\right\}}\left(T,T_{c}\right)\sim T^{-2}\sqrt{1-T^{2}/T_{c}^{2}},$$

hence the scaling of the temporal probability density $P_{k}^{\left\{T\right\}}$ follows the power law with −2 exponent with additional $\sqrt{1-T/T_{c}}$ falloff in close vicinity of the critical point in agreement with temporal scaling of neuronal avalanches reported in [12, 13].

## 6 Multi-mode avalanche probability

The above single wave mode analysis shows that the probability density $P_{k}^{\left\{T\right\}}$ for any arbitrary selected wave mode *k* with arbitrary chosen threshold follows a power law distribution with −2 exponent, therefore, a mixture of multiple wave modes that enters into the spatiotemporal wave field *ψ*(*x*, *t*) with different amplitudes and different thresholds will again show nothing more than the same power law distribution.

**FIGURE 2:**
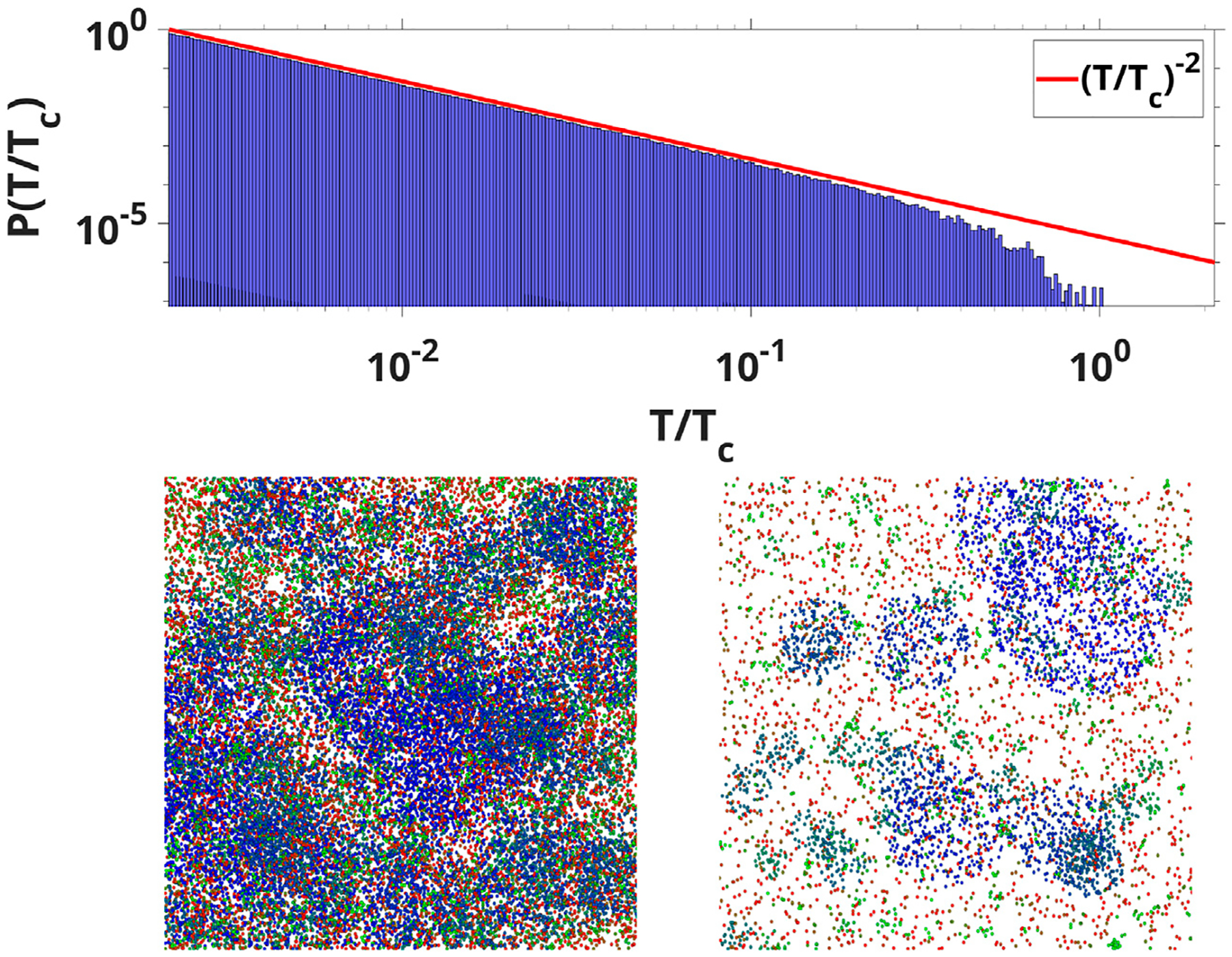
(Top) The avalanche durations distribution for all wave modes compared with the −2 exponent (Bottom) WETCOW modes randomly distributed and propagated on a 1,000 by 1000 grid. Two examples of temporal signal snapshots with different values of signal threshold are shown (color pallet encodes the change of frequencies from the smallest (blue) to the largest (red). Localized regions of wave activity in the spatial domain are clearly evident.

To clearly demonstrate that the probability density function $P_{k}^{\left\{T\right\}}$ of finding a spike reflects the avalanche duration distribution we conducted a simple numerical experiment using a procedure that replicates the original experimental method of computing neuronal avalanches employed in the original papers by Beggs and Plenz [12, 13]. We used 10^6^ wave modes with arbitrary parameters and computed avalanches by cutting the temporal series with a threshold (converting the event to a single dot or “spike”), then binning the signal using a time equal to the average inter-spike interval. After that, an avalanche duration is given by the time between two empty bins. Figure 2 compares the avalanche distribution for all wave modes with the −2 exponent.

Similar proof of equivalence of the single mode probability density function $P_{k}^{\left\{T\right\}}$ and a probability density of a multi-mode avalanche event obtained by the method replicating the experimental demarcation of the quiescence, that we will denote as *p*^*a*^(*T*), can be also derived using simple analytical considerations. The probability $P_{0\leq T^{\prime }\leq T+\Delta T}^{a}$ that an avalanche happens at any time between 0 and *T* + Δ*T*, where Δ*T* is some small binning interval used by the above experimental method, can be expressed as

(33) $$P_{0\leq T^{\prime }\leq T+\Delta T}^{a}=\overset{T+\Delta T}{\underset{0}{\int }}p^{a}\left(T^{\prime }\right)dT^{\prime }=\overset{T}{\underset{0}{\int }}p^{a}\left(T^{\prime }\right)dT^{\prime }+\overset{T+\Delta T}{\underset{T}{\int }}p^{a}\left(T^{\prime }\right)dT^{\prime }\approx P_{0\leq T^{\prime }\leq T}^{a}+p^{a}(T)\Delta T.$$

Since the probability

(34) $$P_{k_{j}}^{\left\{T\right\}}\left(T,T_{c_{j}}\right)\Delta T=v_{j}T^{-2}\sqrt{1-T^{2}/T_{c_{j}}^{2}}\Delta T,$$

(where *ν*_*j*_ is an arbitrary mode specific proportionality constant) describes the probability of finding a signal for a single mode *j* (*j* = 1 … *N*) in a time interval between *T* and *T* + Δ*T*, the probability that the condition for detection of a multi mode avalanche is recorded in the same interval can be expressed as

(35) $$p^{a}(T)\Delta T=\left[1-P_{0}(T-\Delta T)\right]\times P_{0}(T)\;\text{where }P_{0}(T)=\overset{N}{\underset{j=1}{\prod }}\left(1-P_{k_{j}}^{\left\{T\right\}}\left(T,T_{c_{j}}\right)\Delta T\right)$$

where all wave modes are assumed to be independent. The second factor *P*_0_*T*) represents the probability that there is no signal for any of the modes detected between *T* and *T* + Δ*T*. The first factor (1 − *P*_0_ (*T* − Δ*T*)) makes sure that no avalanche was recorded in the previous Δ*T* bin, that is a signal for at least one mode has been found in the interval between *T* − Δ*T* and *T*.

An expansion of Eq. 35 in the leading order of Δ*T* gives for the avalanche probability density *p*^*a*^(*T*)

(36) $$p^{a}(T)\approx T^{-2}\overset{N}{\underset{j=1}{\sum }}v_{j}\sqrt{1-T^{2}/T_{c_{j}}^{2}},$$

that is the avalanche probability density *p*^*a*^(*T*) shows the same *T*^−2^ scaling as the probability density of finding signal for a single mode.

If additionally the criticality parameters $T_{c_{j}}$ for all wave modes *k*_*j*_ are assumed to be the same $\left(T_{c_{j}}\equiv T_{c}\right)$ then the avalanche probability density scaling takes exactly the same form as the single mode probability density

(37) $$p^{a}(T)\sim P_{k}^{\left\{T\right\}}(T)\sim T^{-2}\sqrt{1-T^{2}/T_{c}^{2}}.$$

## 7 Spatial spike detection probability

Due to the reciprocity of the temporal and spatial representations of the Hamiltonian form Eq. 1 equations for the spatial wave amplitude have the same form as the temporal equations Eq. 21 and Eq. 22

(38) $$\frac{d\tilde{A}}{d\xi }=\tilde{A}+{\tilde{A}}^{2}\left[R_{a}\text{ cos}(\phi -\Phi )-\alpha \right],$$

(39) $$\frac{d\phi }{d\xi }=\tilde{k}+\tilde{A}R_{\phi }\text{ cos }\phi ,$$

under similar scaling (the spatial equivalent of Eq. 11) of the wave amplitude, the coordinate, and the wave number

(40) $$A=\lambda \tilde{A},\;x=\frac{\xi }{\lambda },\;k=\tilde{k}\lambda .$$

In the spatial domain, this leads to the critical parameters ${\tilde{A}}_{c}$ and. ${\tilde{k}}_{c}$

(41) $${\tilde{k}}_{c}=\frac{R_{\phi }\text{ cos }\phi_{c}}{\alpha +R_{a}\text{ cos}\left(\phi_{c}+\Phi \right)},\;{\tilde{A}}_{c}={\tilde{k}}_{c}/R_{\phi },$$

(42) $$\phi_{c}=\text{arctan}\left[\frac{R_{a}\text{ sin Φ}}{\sqrt{\alpha^{2}-{\left(R_{a}\text{ sin Φ}\right)}^{2}}}\right],$$

Although our simple one dimensional scaling estimates do not take into account the intrinsic spatial scales of the brain, e.g., cortex radius of curvature, cortical thickness, *etc*., nevertheless, even in this simplified form the similarity between spatial and temporal non-linear equations suggests that the non-linear spatial wave behavior will generally look like spiking in the spatial domain where some localized regions of activity are separated by areas that are relatively signal free and this separation will increase near the critical point. Exactly this behavior was reported in the original experimental studies of the neuronal avalanches [12, 13], where it was stated that the analysis of the contiguity index revealed that activity detected at one electrode is most often skipped over the nearest neighbors. Interestingly, this experimental observation of near critical non-linear waves was presented as an indicator that the activity propagation is not wave-like. But we see here that they are directly explained within the context of the WETCOW wave model. Of significant practical importance will be the effects of the intrinsic spatial scales of the brain that will certainly affect the details of the spatial critical wave dynamics and so their inclusion will be important for more completely characterizing the details of brain criticality and will be the focus of future investigations.

Using the spatial equations Eq. 38 and Eq. 39 similar scaling results can be obtained for the wave number *k* and the linear spatial dimension *L* probabilities for every wave mode with the frequency *ω* as.

(43) $$P_{\omega }^{\left\{k\right\}}\left(k,k_{c}\right)\sim k^{-1}\sqrt{k^{2}/k_{c}^{2}-1},$$

(44) $$P_{\omega }^{\left\{L\right\}}\left(L,L_{c}\right)\sim L^{-2}\sqrt{1-L^{2}/L_{c}^{2}},$$

where *L* is the linear spatial scale related to the wave number as *k* = 2*π*/*L*.

The linear spatial dimension of the avalanche *L* is related to its area *S* on a 2 dimensional surface as $L=\sqrt{S}$, hence

(45) $$\overset{L_{c}}{\underset{0}{\int }}P_{\omega }^{\left\{L\right\}}\left(L,L_{c}\right)dL=\overset{S_{c}}{\underset{0}{\int }}\frac{P_{\omega }^{\left\{L\right\}}\left(\sqrt{S},\sqrt{S_{c}}\right)}{2\sqrt{S}}dS\;=\overset{S_{c}}{\underset{0}{\int }}P_{\omega }^{\left\{S\right\}}\left(S,S_{c}\right)dS,$$

(46) $$P_{\omega }^{\left\{S\right\}}\left(S,S_{c}\right)\sim S^{-3/2}\sqrt{1-S/S_{c}},$$

hence the spatial probability scaling for the size *S* follows the power law with −3/2 exponent again with additional $\sqrt{1-S/S_{c}}$ falloff in close vicinity of the critical point, that is also in agreement with experimentally reported spatial scaling of neuronal avalanches [12, 13]. We would like to mention that the non-linear anharmonic oscillations described by the (21) and (22) only exists for frequencies and wave numbers that are above the critical frequency *ω*_*c*_ or the critical wave number *k*_*c*_ values that define maximal possible temporal *T*_*c*_ or spatial *L*_*c*_ scales of the non-linear oscillations. If the finite system sizes are below those maximal values the cutoffs will be defined by the system scales.

We would like emphasize again the generality of our analysis that makes no assumptions about parameters used in Hamiltonian form Eq. 1, and hence in the equations Eq. 21 and Eq. 22 or (38) and (39), analytically deriving scaling valid for a wide (and arbitrary) range of those parameters. This is in striking difference from analyses and results based on oversimplified *ad hoc* numerical studies of synchronization in networks [36, 38]. Those typical numerical analysis studies consider networks of *completely identical* individual nodes sometimes even *globally* connected with *completely identical* weights. Therefore, all these studies require artificial (and significantly high levels of) noise added to each node just to be able to impose some range of scales into the system. This is an artificial and, as demonstrated here, unnecessary complication. The consequence of such models is that they are capable of obtaining something that resembles scale free behavior with exponent values that are in general rather vague and strongly noise dependent. Without this sufficiently strong noise those studies of course are not capable to show any scale free behavior. It is essential to realize that such models are thus highly dependent on the noise properties, and less so on the actual properties of the brain tissue itself as in the WETCOW theory, which is the critical link to practical applications of any brain activity theory. By contrast, no externally induced stochasticity in the form of additional noise term is required for our analysis.

Another important point is that for deriving scale free exponents in our approach we do not require to know the details of the coupling between nodes, essentially viewing all nodes as completely non-interacting. Presence of interactions in the form of (9) will not modify our analysis, and will not require any of the common *ad hoc* assumptions of identical global coupling between nodes [38]. When coupling between some of the nodes in (9) is sufficiently strong and these nodes are completely synchronized, we can always replace this subset of completely synchronized nodes by a single node and continue again with the same presented in this paper “coupling-independent” node analysis.

## 8 Effects of criticality on spike length

The assumption of the fixed spike duration *δt*_*s*_ used in Eq. 30 and 32 (or the spike length for spatial spiking in Eq. 43 and Eq. 44) can be improved by estimating the scaling of the spike width as a function of the criticality parameter from the amplitude equation (we will use the temporal form of the equation Eq. 21 but the spatial analysis with equation Eq. 38 is exactly the same).

Dividing equation Eq. 21 by *Ã* and taking an integral around some area in the vicinity of the amplitude peak *Ã*_*max*_ we can write

(47) $$\overset{{\tilde{A}}_{+}}{\underset{A^{-}}{\int }}\frac{1}{\tilde{A}}d\tilde{A}=\overset{\tau_{+}}{\underset{\tau_{-}}{\int }}d\tau \overset{\Phi_{+}}{\underset{\Phi_{-}}{\int }}\frac{{\tilde{\omega }}_{c}}{R_{\phi }}\frac{R_{a}\text{ cos}(\phi -\Phi )-\alpha }{\tilde{\omega }+{\tilde{\omega }}_{c}\text{ cos }\phi }d\phi ,$$

where *τ*_±_ = *τ*_*max*_ ± *δτ*, and *τ*_*max*_ is the location of spiking peak. Neglecting the spike shape asymmetries, i.e., assuming that *τ*_±_ correspond to symmetric changes in both the amplitudes *Ã*_±_ = *Ã*(*τ*_±_) = *Ã*_*max*_ − *δÃ*, and the phases Φ_±_ = Φ(*τ*_±_) = Φ ± *δ*Φ, we can then estimate the spike duration *δt*_*s*_ ≡ (*τ*_+_ − *τ*_−_)/*γ* as

(48) $$\delta t_{s}=\frac{1}{\gamma }\overset{\Phi +\delta \Phi }{\underset{\Phi -\delta \Phi }{\int }}\frac{1-R(\text{cos}(\Phi )+\text{cos}(\phi -\Phi ))}{\tilde{\omega }+{\tilde{\omega }}_{c}\text{ cos }\phi }d\phi ,$$

where, similar to the spiking period estimation in Eq. 28, we again assume that the main contribution comes from the change of the oscillation phase, hence *Ã*_*c*_ can be substituted for *Ã*. For *δ*Φ some fixed value that is smaller or around a quarter of the period (i.e., *δ*Φ ≲ *π*/2) can be chosen, and $R={\tilde{\omega }}_{c}R_{a}/R_{\phi }$.

**FIGURE 3:**
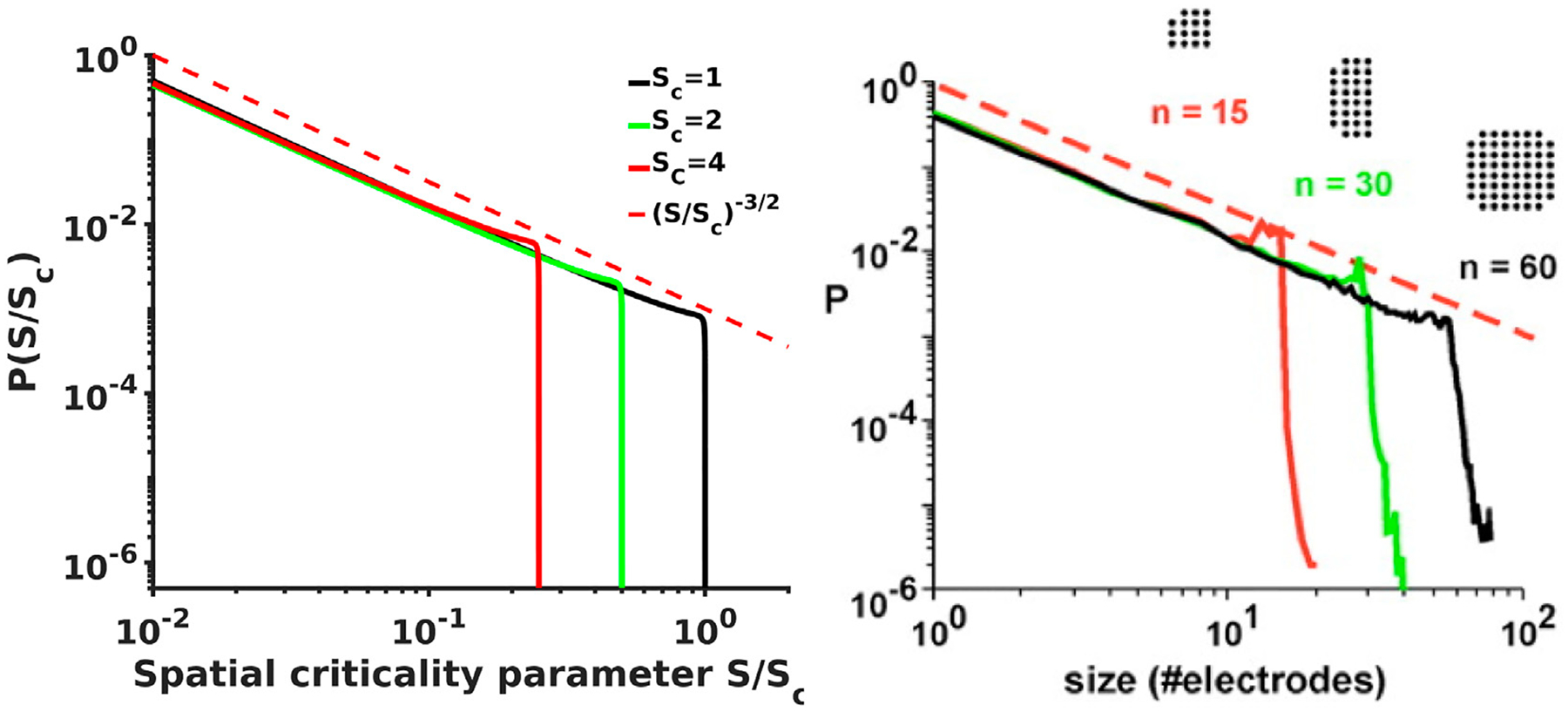
[Left] Analytical probability density spectra as a function of brain waves criticality parameter *S*/*S*_*c*_ show excellent agreement with the experimental avalanche data [Right, from [12, 13]] reproducing not only the overall shape of the spectra with the −3/2 power exponent at the initial scale free part of the spectra and the steep falling edge in the vicinity of the critical point, but also reproduce the fine details such as the small bump-like flattening of the spectra at the transition from −3/2 leg to the steep falling edge that is clearly evident in experimental spectra.

**FIGURE 4:**
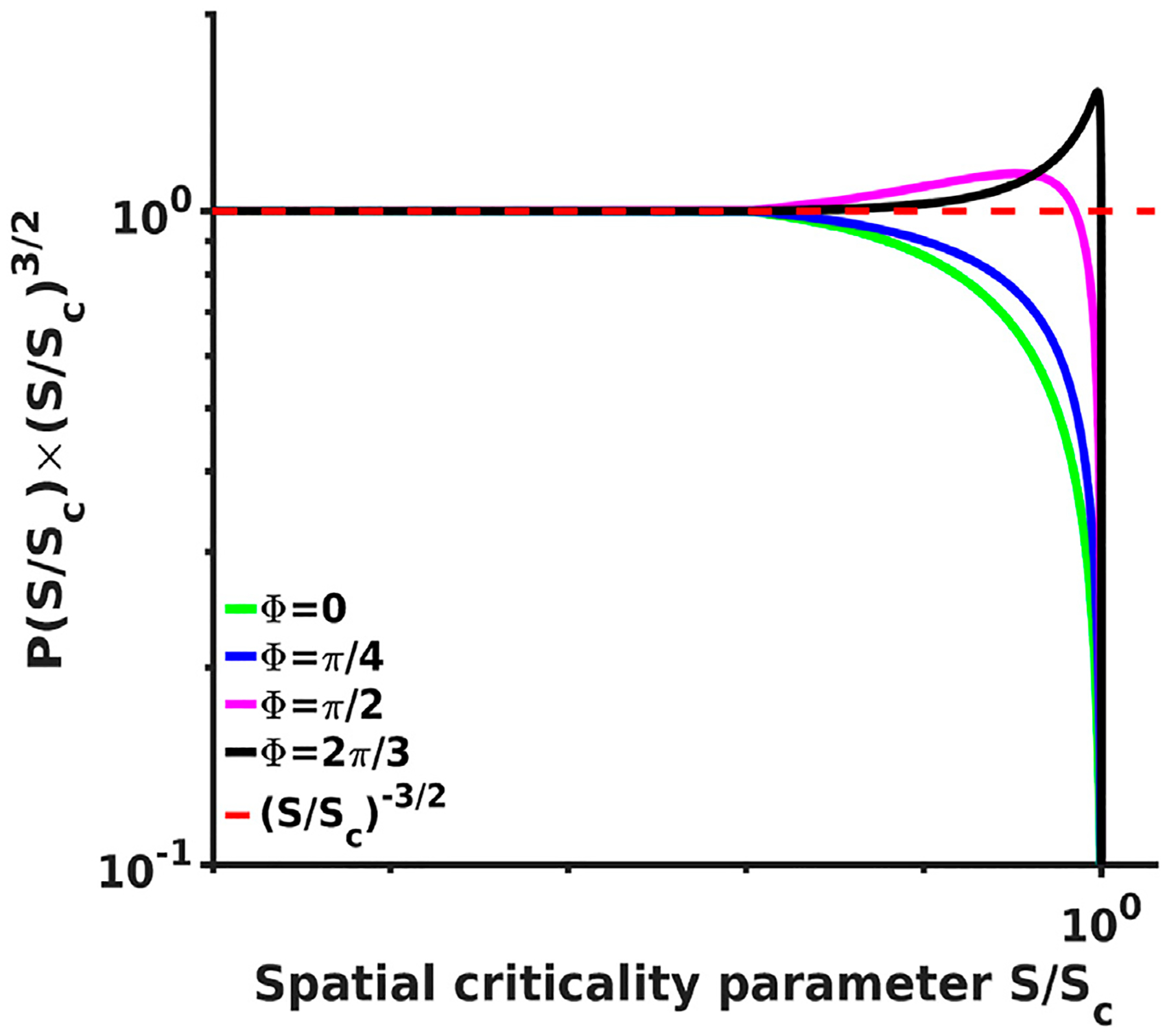
Analytical probability density spectra multiplied by a (*S*/*S*_*c*_)^3/2^ as a for several function of brain waves criticality parameter *S*/*S*_*c*_ plotted values of the phase shift Φ.

An expression (48) can be evaluated in closed form but we do not include it here and instead plotted the final spatial probability density spectra *P*(*S*/*S*_*c*_), similarly obtained from the expression for *δl*_*s*_/*L*_*s*_ again substituting $L=\sqrt{S}$ and $dL=dS/(2\sqrt{S})$, for several values of the *S*_*c*_ parameter (Figure 3), as well as for several values of the phase shift Φ (Figure 4). The spectra clearly show again the same power law dependence with −3/2 exponent as was reported in [12, 13] followed by a steep falloff sufficiently close to the critical point. What is interesting, however, is that the spectra for Φ = *π*/2 (and this is the phase shift value used for spiking solutions reported in [1, 2, 39]) recover even the fine structure of the scaling and clearly show the small bump at the end of the scale free part of the spectra where the local probability deflects from the initial −3/2 power exponent and flattens first before turning in to the steep falloff. These small bumps are evident in all experimental spectra [12, 13] shown in Figure 3 as well.

## 9 Conclusion

Brain activity in general and neuronal avalanches in particular show an abundance of very complex and strangely organized activity patterns. Understanding the nature and the origin of cascades of synchronized activity in the cortex has multiple implications to understanding of organization of cortical functioning. Although originally neuronal avalanches were detected *in vitro* using multi-electrode arrays in 2D slices of cultivated cortex cultures [12, 13], there are now multiple experimental data of *in vivo* avalanche recordings [45–47] involving optical recordings as well [48].

**FIGURE 5:**
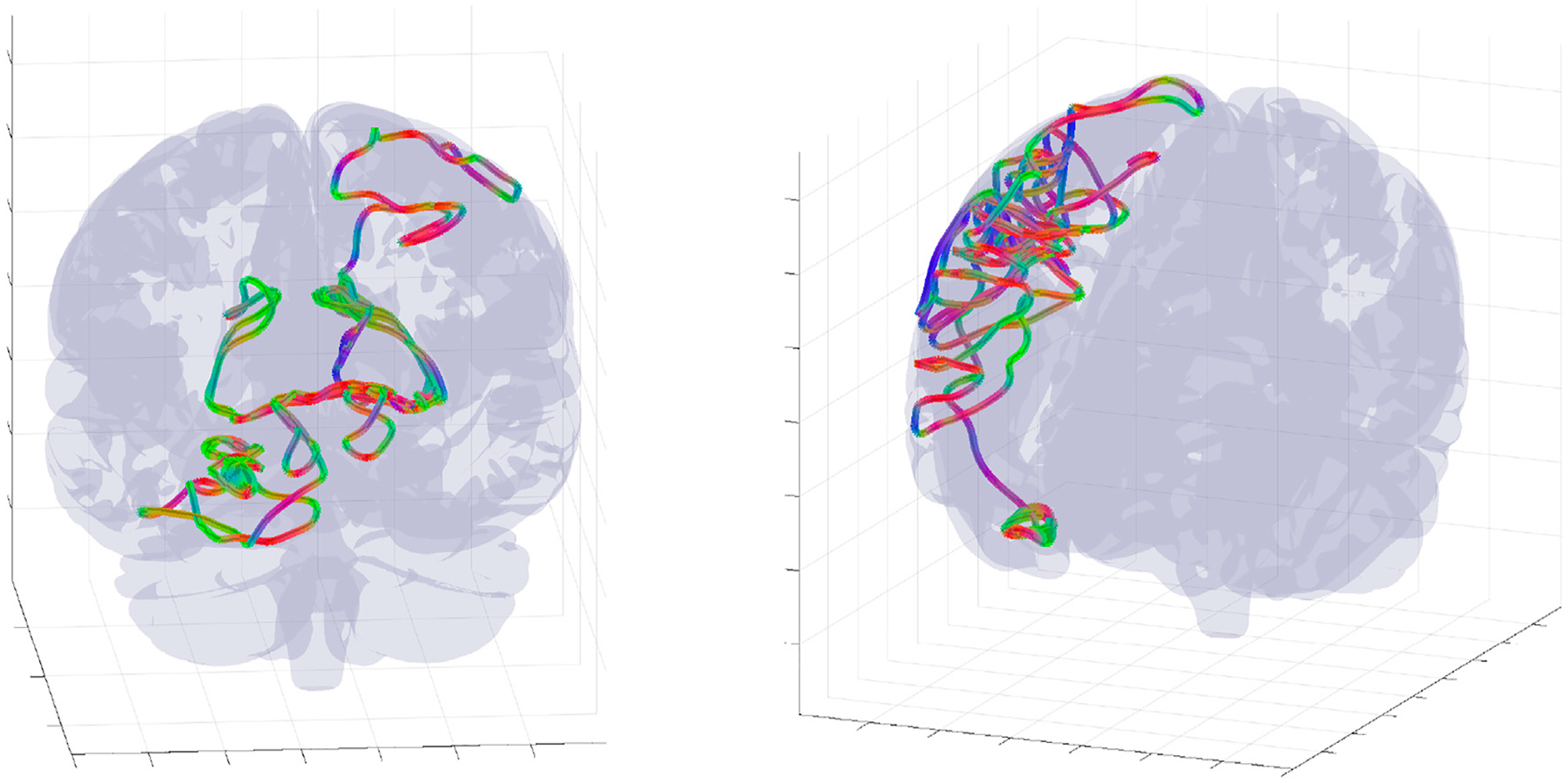
Examples of complete wave mode trajectory snapshots for two randomly selected parameters and initial conditions. The trajectories was randomly selected from an ensemble of 10^6^ WETCOW modes used for generation of probability distributions of Figure 6.

**FIGURE 6:**
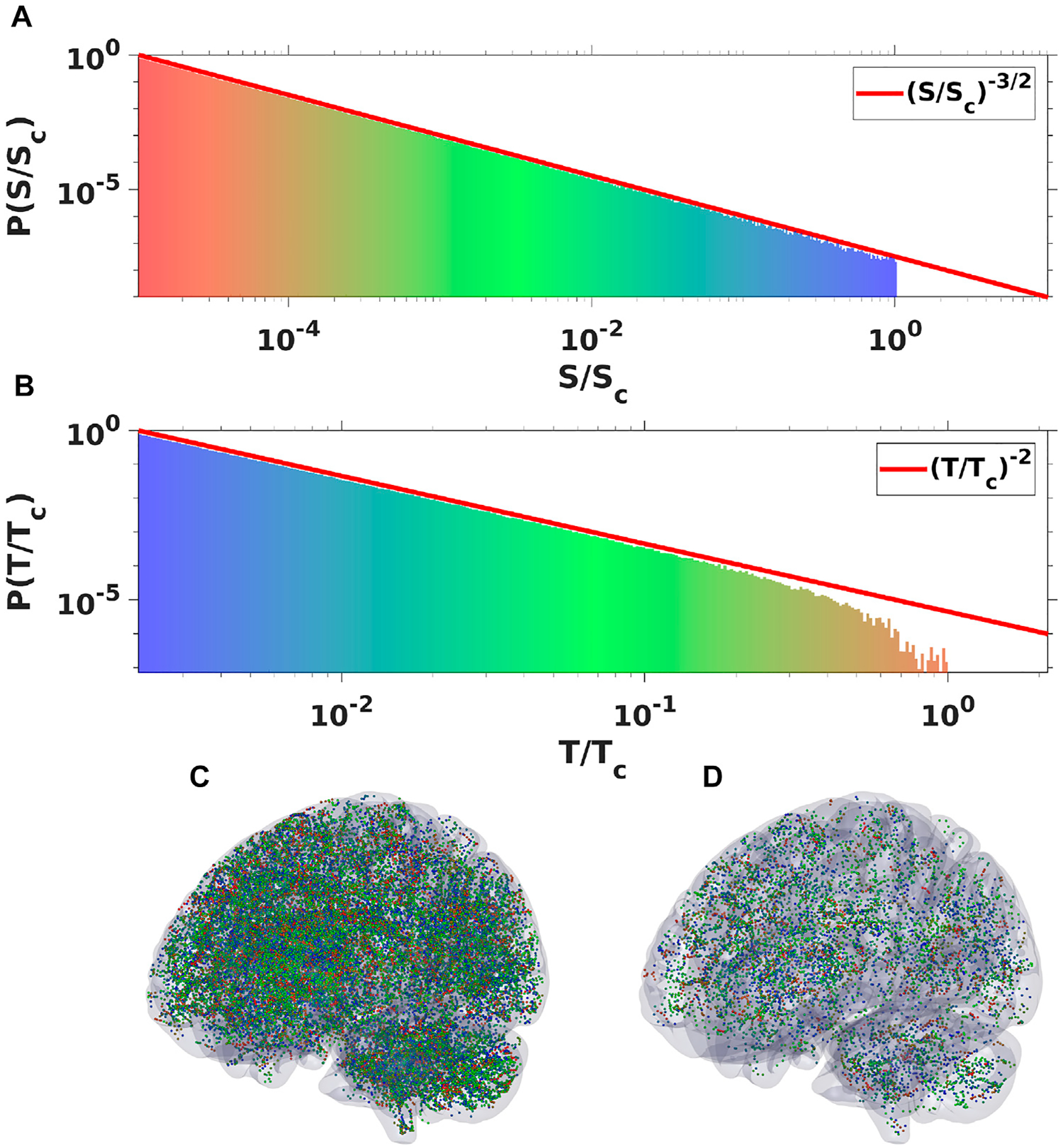
Plots of spatial (A) and temporal (B) probability density spectra obtained by binning oscillatory signal of ensemble of 10^6^ WETCOW modes randomly distributed and propagated through cortical tissue. Two examples of temporal signal (dots or “spikes”) snapshots with different values of signal threshold are shown in (C) and (D) (color pallet encodes the change of frequencies from the smallest (blue) to the largest (red).

One of the properties of the WETCOW wave modes is that the anisotropy structure of brain conductivity as well as the structure of brain inhomogeneity favors their propagation in the outer regions of the cortex (see, for example, Figure 2 of [1, 2]). Neuronal avalanches are measured in the most external layer of the cortex and, usually, introducing the electrodes deeper in the cortical columns will eliminate the scale-free distributions. Therefore, it seems to be an interesting problem to check the whole-brain scale free distribution in the region of typical propagation of WETCOW wave modes. To do this numerical experiment we generated an ensemble of 10^6^ WETCOW modes distributed and randomly propagating through inhomogeneous and anisotropic cortical tissue. Figure 5 shows two randomly selected snapshots of wave mode trajectories that were generated using the procedure described in details in [1] and propagate in the surface-like 2D manner in the external layer of the cortex. Using the same procedure, that replicates the original experimental neuronal avalanche detection method, that is thresholding and then binning the wave signal into dots or “spikes”, we again see that the WETCOW modes show scale free behavior as shown in Figure 6.

In summary, in this paper we have presented an analysis of temporal and spatial probability density spectra that are generated due to the critical dynamics of the non-linear weakly evanescent cortical wave (WETCOW) modes [1, 2]. The Hamiltonian framework developed for these WETCOW modes in [39] is advantageous in that it explicitly uncovers the reciprocity of the temporal and the spatial dynamics of the evolutionary equations. Therefore, in the non-linear regime sufficiently close to the critical point the spatial behavior of the wave modes displays features similar to the properties of their non-linear temporal dynamics that can be described as spatial domain spiking, with localized regions of wave activity separated by quiescent areas, with this spatial spiking intermittence increasing near the critical point. Similar spatial behavior was observed experimentally in neuronal avalanches, when activity detected at one electrode was typically skipped over the nearest neighbors. This was interpreted as evidence that avalanche spatial intermittency is not wave-like in nature [12, 13]. Our results demonstrate the contrary, however: the spatial patterns are the direct result of non-linear interactions of weakly evanescent cortical waves.

Both temporal and spatial scaling expressions analytically estimated from the non-linear amplitude/phase evolutionary equations show excellent agreement with the experimental neuronal avalanche probability spectra reproducing not only the general average power law exponent values and falloffs in the vicinity of the critical point, but also finding some very subtle but nevertheless clearly experimentally evident fine details, like bumps in the transition region at the edge of the scale free part of the probability spectra.

In a more general way these results may be applicable not only to neuronal avalanches but to many other physical systems that involve wave processes as they show that a system of wave modes interacting through all possible combinations of the third order non-linear terms described by a general wave Hamiltonian necessarily produces anharmonic wave modes with temporal and spatial scaling properties that follow scale free power laws.

## Data Availability

The original contributions presented in the study are included in the article/Supplementary Material, further inquiries can be directed to the corresponding author.

## References

[1] Galinsky VL, Frank LR. Universal theory of brain waves: From linear loops to nonlinear synchronized spiking and collective brain rhythms. Phys Rev Res (2020) 2: 023061. (1–23). doi:10.1103/physrevresearch.2.023061 33718881 PMC7951957

[2] Galinsky VL, Frank LR. Brain waves: Emergence of localized, persistent, weakly evanescent cortical loops. J Cogn Neurosci (2020) 32:2178–202. doi:10.1162/jocn_a_01611 32692294 PMC7541648

[3] Buzsaki G Rhythms of the brain. Oxford: Oxford University Press (2006).

[4] Hodgkin AL, Huxley AF. A quantitative description of membrane current and its application to conduction and excitation in nerve. J Physiol (Lond.) (1952) 117:500–44. doi:10.1113/jphysiol.1952.sp004764 12991237 PMC1392413

[5] Fitzhugh R. Impulses and physiological states in theoretical models of nerve membrane. Biophys J (1961) 1:445–66. doi:10.1016/s0006-3495(61)86902-6 19431309 PMC1366333

[6] Nagumo J, Arimoto S, Yoshizawa S. An active pulse transmission line simulating nerve axon. Proc IRE (1962) 50:2061–70. doi:10.1109/jrproc.1962.288235

[7] Morris C, Lecar H. Voltage oscillations in the barnacle giant muscle fiber. Biophys J (1981) 35:193–213. doi:10.1016/s0006-3495(81)84782-0 7260316 PMC1327511

[8] Izhikevich EM. Simple model of spiking neurons. IEEE Trans Neural Netw (2003) 14:1569–72. doi:10.1109/tnn.2003.820440 18244602

[9] Gerstner W, Kistler WM, Naud R, Paninski L. Neuronal dynamics: From single neurons to networks and models of cognition. New York, NY, USA: Cambridge University Press (2014).

[10] Kulkarni A, Ranft J, Hakim V. Synchronization, stochasticity, and phase waves in neuronal networks with spatially-structured connectivity. Front Comput Neurosci (2020) 14:569644. doi:10.3389/fncom.2020.569644 33192427 PMC7604323

[11] Kim R, Sejnowski TJ. Strong inhibitory signaling underlies stable temporal dynamics and working memory in spiking neural networks. Nat Neurosci (2021) 24: 129–39. doi:10.1038/s41593-020-00753-w 33288909

[12] Beggs JM, Plenz D. Neuronal avalanches in neocortical circuits. J Neurosci (2003) 23:11167–77. doi:10.1523/jneurosci.23-35-11167.2003 14657176 PMC6741045

[13] Beggs JM, Plenz D. Neuronal avalanches are diverse and precise activity patterns that are stable for many hours in cortical slice cultures. J Neurosci (2004) 24:5216–29. doi:10.1523/jneurosci.0540-04.2004 15175392 PMC6729198

[14] Plenz D, Ribeiro TL, Miller SR, Kells PA, Vakili A, Capek EL. Self-organized criticality in the brain. Front Phys (2021) 9:639389. doi:10.3389/fphy.2021.639389

[15] Friedman N, Ito S, Brinkman BA, Shimono M, DeVille RE, Dahmen KA, Universal critical dynamics in high resolution neuronal avalanche data. Phys Rev Lett (2012) 108:208102. doi:10.1103/physrevlett.108.208102 23003192

[16] Chialvo DR. Emergent complex neural dynamics. Nat Phys (2010) 6:744–50. doi:10.1038/nphys1803

[17] Beggs JM, Timme N. Being critical of criticality in the brain. Front Physiol (2012) 3:163. doi:10.3389/fphys.2012.00163 22701101 PMC3369250

[18] Priesemann V, Wibral M, Valderrama M, Pröpper R, Le Van Quyen M, Geisel T, Spike avalanches in vivo suggest a driven, slightly subcritical brain state. Front Syst Neurosci (2014) 8:108. doi:10.3389/fnsys.2014.00108 25009473 PMC4068003

[19] Cramer B, Stöckel D, Kreft M, Wibral M, Schemmel J, Meier K, Control of criticality and computation in spiking neuromorphic networks with plasticity. Nat Commun (2020) 11:2853. doi:10.1038/s41467-020-16548-3 32503982 PMC7275091

[20] Fontenele AJ, de Vasconcelos NAP, Feliciano T, Aguiar LAA, Soares-Cunha C, Coimbra B, Criticality between cortical states. Phys Rev Lett (2019) 122:208101. doi:10.1103/physrevlett.122.208101 31172737

[21] Allegrini P, Paradisi P, Menicucci D, Laurino M, Piarulli A, Gemignani A. Self-organized dynamical complexity in human wakefulness and sleep: Different critical brain-activity feedback for conscious and unconscious states. Phys Rev E Stat Nonlin Soft Matter Phys (2015) 92:032808. doi:10.1103/physreve.92.032808 26465529 PMC4909144

[22] Allegrini P, Menicucci D, Bedini R, Fronzoni L, Gemignani A, Grigolini P, Spontaneous brain activity as a source of ideal1/fnoise. Phys Rev E Stat Nonlin Soft Matter Phys (2009) 80:061914. doi:10.1103/physreve.80.061914 20365197

[23] Teixeira Borges AF, Irrmischer M, Brockmeier T, Smit DJA, Mansvelder HD, Linkenkaer-Hansen K. Scaling behaviour in music and cortical dynamics interplay to mediate music listening pleasure. Sci Rep (2019) 9:17700. doi:10.1038/s41598-019-54060-x 31776389 PMC6881362

[24] Fosque LJ, Williams-García RV, Beggs JM, Ortiz G. Evidence for quasicritical brain dynamics. Phys Rev Lett (2021) 126:098101. doi:10.1103/physrevlett.126.098101 33750159

[25] Bak P, Tang C, Wiesenfeld K. Self-organized criticality: An explanation of the 1/f noise. Phys Rev Lett (1987) 59:381–4. doi:10.1103/PhysRevLett.59.381 10035754

[26] Bak P, Tang C, Wiesenfeld K. Self-organized criticality. Phys Rev A (1988) 38: 364–74. doi:10.1103/PhysRevA.38.364 9900174

[27] Zapperi S, Lauritsen KB, Stanley HE. Self-organized branching processes: Mean-field theory for avalanches. Phys Rev Lett (1995) 75:4071–4. doi:10.1103/PhysRevLett.75.4071 10059807

[28] Bækgaard Lauritsen K, Zapperi S, Stanley HE. Self-organized branching processes: Avalanche models with dissipation. Phys Rev E (1996) 54:2483–8. doi:10.1103/PhysRevE.54.2483 9965358

[29] Eurich CW, Herrmann JM, Ernst UA. Finite-size effects of avalanche dynamics. Phys Rev E Stat Nonlin Soft Matter Phys (2002) 66:066137. doi:10.1103/physreve.66.066137 12513377

[30] Bédard C, Kröger H, Destexhe A. Does the 1/*f* frequency scaling of brain signals reflect self-organized critical states? Phys Rev Lett (2006) 97:118102. doi:10.1103/PhysRevLett.97.118102 17025932

[31] Touboul J, Destexhe A. Can power-law scaling and neuronal avalanches arise from stochastic dynamics? PLoS One (2010) 5:e8982. doi:10.1371/journal.pone.0008982 20161798 PMC2820096

[32] Touboul J, Destexhe A. Power-law statistics and universal scaling in the absence of criticality. Phys Rev E (2017) 95:012413. doi:10.1103/physreve.95.012413 28208383

[33] Robinson PA, Rennie CJ, Wright JJ. Propagation and stability of waves of electrical activity in the cerebral cortex. Phys Rev E (1997) 56:826–40. doi:10.1103/PhysRevE.56.826

[34] Yang DP, Robinson PA. Critical dynamics of Hopf bifurcations in the corticothalamic system: Transitions from normal arousal states to epileptic seizures. Phys Rev E (2017) 95:042410. doi:10.1103/physreve.95.042410 28505725

[35] Robinson PA, Rennie CJ, Rowe DL. Dynamics of large-scale brain activity in normal arousal states and epileptic seizures. Phys Rev E Stat Nonlin Soft Matter Phys (2002) 65:041924. doi:10.1103/physreve.65.041924 12005890

[36] di Santo S, Villegas P, Burioni R, Muñoz MA. Landau–Ginzburg theory of cortex dynamics: Scale-free avalanches emerge at the edge of synchronization. Proc Natl Acad Sci (2018) 115:E1356–E1365. doi:10.1073/pnas.1712989115 29378970 PMC5816155

[37] Gireesh ED, Plenz D. Neuronal avalanches organize as nested theta- and beta/gamma-oscillations during development of cortical layer 2/3. Proc Natl Acad Sci (2008) 105:7576–81. doi:10.1073/pnas.0800537105 18499802 PMC2396689

[38] Buendía V, Villegas P, Burioni R, Muñoz MA. Hybrid-type synchronization transitions: Where incipient oscillations, scale-free avalanches, and bistability live together. Phys Rev Res (2021) 3:023224. doi:10.1103/PhysRevResearch.3.023224

[39] Galinsky VL, Frank LR. Collective synchronous spiking in a brain network of coupled nonlinear oscillators. Phys Rev Lett (2021) 126:158102. doi:10.1103/PhysRevLett.126.158102 33929245 PMC8095823

[40] Ott E, Antonsen TM. Long time evolution of phase oscillator systems. Chaos (2009) 19:023117. doi:10.1063/1.3136851 19566252

[41] Tyulkina IV, Goldobin DS, Klimenko LS, Pikovsky A. Dynamics of noisy oscillator populations beyond the ott-antonsen ansatz. Phys Rev Lett (2018) 120:264101. doi:10.1103/PhysRevLett.120.264101 30004770

[42] Kuramoto Y Chemical oscillations, waves, and turbulence. Heidelberg: Springer Berlin Heidelberg (2013). Dover Books on Chemistry Series (Dover Publications, Incorporated).

[43] Daido H Generic scaling at the onset of macroscopic mutual entrainment in limit-cycle oscillators with uniform all-to-all coupling. Phys Rev Lett (1994) 73:760–3. doi:10.1103/physrevlett.73.760 10057530

[44] Crawford JD. Scaling and singularities in the entrainment of globally coupled oscillators. Phys Rev Lett (1995) 74:4341–4. doi:10.1103/physrevlett.74.4341 10058476

[45] Petermann T, Thiagarajan TC, Lebedev MA, Nicolelis MA, Chialvo DR, Plenz D. Spontaneous cortical activity in awake monkeys composed of neuronal avalanches. Proc Natl Acad Sci U S A (2009) 106:15921–6. doi:10.1073/pnas.0904089106 19717463 PMC2732708

[46] Bellay T, Shew WL, Yu S, Falco-Walter JJ, Plenz D. Selective participation of single cortical neurons in neuronal avalanches. Front Neural Circuits (2020) 14:620052. doi:10.3389/fncir.2020.620052 33551757 PMC7862716

[47] Hahn G, Petermann T, Havenith MN, Yu S, Singer W, Plenz D, Neuronal avalanches in spontaneous activity *in vivo*. J Neurophysiol (2010) 104:3312–22. doi:10.1152/jn.00953.2009 20631221 PMC3007625

[48] Frostig RD, Chen-Bee CH, Johnson BA, Jacobs NS. Imaging cajal’s neuronal avalanche: How wide-field optical imaging of the point-spread advanced the understanding of neocortical structure-function relationship. Neurophotonics (2017) 4:031217. doi:10.1117/1.nph.4.3.031217 28630879 PMC5467767

